# NUPR1 as a central stress-adaptation node in cancer: integrating metabolic rewiring, cell death, and therapy resistance

**DOI:** 10.1186/s12929-026-01254-x

**Published:** 2026-05-09

**Authors:** Tanqing Long, Qi Wang, Yinpeng Le, Mi Zhang, Yan Sun, Juan Iovanna, Lei Li, Chuanrui Xu, Juan Liu

**Affiliations:** 1 School of Pharmacy, Tongji Medical College, Huazhong University of Science and Technology, Wuhan, 430030 China; 2 Hepato-Pancreato-Biliary Center, Beijing Tsinghua Changgung Hospital, Key Laboratory of Digital Intelligence Hepatology (Ministry of Education), School of Clinical Medicine, Tsinghua Medicine, Tsinghua University, Beijing, 102218 China; 3 Zhejiang-Mauritius Joint Research Center for Biomaterials and Tissue Engineering, School of Materials Science and Engineering, Zhejiang Sci-Tech University, Hangzhou, 310018 China; 4 Centre de Recherche en Cancérologie de Marseille (CRCM), INSERM U1068, CNRS UMR 7258, Aix-Marseille Université and Institut Paoli-Calmettes, Parc Scientifique et Technologique de Luminy, Translational Research and Therapeutic Targets in Pancreatic Cancer, Marseille, France

**Keywords:** NUPR1, Cell death, Metabolic reprogramming, Tumor microenvironment, Therapeutic resistance

## Abstract

Nuclear protein 1 (NUPR1) is an intrinsically disordered, stress-adaptive regulator that sits at the intersection of transcriptional plasticity and proteostatic control, broadly upregulated across malignancies and tightly associated with poor prognosis. Here, we synthesize evidence positioning NUPR1 as a central node of tumor adaptation that integrates metabolic rewiring, proteostatic balance, and cell-death checkpoints into a unified stress-response framework. NUPR1 orchestrates lipogenic and glycolytic programs, sustains lysosomal biogenesis and autophagic flux, and governs cell-fate decisions by restraining apoptosis and ferroptosis through iron and redox control. Beyond tumor-intrinsic roles, NUPR1 remodels the tumor microenvironment by driving immunosuppressive macrophage polarization and amplifying inflammatory signaling, collectively sustaining a pro-survival niche. These circuits underpin broad therapeutic resistance across modalities, spanning chemotherapy, targeted agents, endocrine therapy, and immune checkpoint blockade. We further discuss the development of small-molecule NUPR1 antagonists—including ZZW-115 and emerging chemotypes—that disrupt nuclear trafficking and stress tolerance, alongside formulation strategies to optimize pharmacodynamic potency and safety. Together, these insights establish NUPR1 as a druggable stress-response node and provide a mechanistic framework to overcome resistance and refine adaptive cancer therapy paradigms.

## Introduction

Nuclear protein 1 (NUPR1), located on human chromosome 16p11.2, encodes two distinct protein isoforms. The longer isoform, NUPR1a, comprises 100 amino acids—18 more than NUPR1b—although its precise biological function remains unclear. Most current studies focus on NUPR1b, an 82-amino acid protein with an approximate molecular weight of 8.8 kDa. Its C terminus harbors a classical nuclear localization signal featuring a helix–loop–helix motif enriched in basic residues and containing two conserved acetylation and methylation sites whose acetylation status is essential for nuclear translocation. The N terminus contains a proline-, glutamic acid-, serine- and threonine-rich (PEST) sequence that mediates ubiquitin–proteasome–dependent degradation and post-translational modification of NUPR1 [24, 67, 75].

NUPR1 is an intrinsically disordered protein (IDP) that lacks stable secondary and tertiary structures [24, 91, 103, 109]. As an IDP lacking a stable folded conformation, NUPR1 dynamically responds to cellular stress by forming transient complexes with diverse molecular partners, thereby executing highly versatile regulatory functions [18, 21, 22, 104]. Since its discovery as a stress-inducible factor during acute pancreatitis by Iovanna and colleagues in 1997 [67], subsequent studies by Anne Hansen Ree and others have progressively revealed NUPR1 as a central regulator in tumor biology [75]. NUPR1 exerts dual regulatory functions: (i) directly acting as a transcriptional regulator that binds AT-rich promoter regions (< 300 bp); and (ii) indirectly modulating downstream pathways through key signaling mediators, including NF-κB and SMA/Mothers Against Decapentaplegic (SMAD). Across diverse physiological and pathological contexts—particularly during tumor initiation and progression—NUPR1 orchestrates stress responses, survival–apoptosis balance, autophagy, and metabolic homeostasis. Its major functions can be categorized as follows: (i) Tumor microenvironment regulation—NUPR1 drives Alternatively activated macrophage (M2) polarization within the immunosuppressive milieu [7, 59, 117]; (ii) Metabolic reprogramming—the NUPR1/Sterol regulatory element–binding protein 1 (SREBP1) axis promotes lipogenesis [100], while the NUPR1/Granulin (GRN)–PI3-kinase (PI3K)/Protein kinase B (AKT) pathway enhances glycolysis [52]; (iii) Cell death control—NUPR1 activates the Reticuloendotheliosis viral oncogene homolog B (RelB)-Immediate early response 3 (IER3) antiapoptotic axis [23], upregulates Lipocalin-2 (LCN2)/Ferritin heavy chain 1 (FTH1) to suppress ferroptosis [119], and maintains calcium and energy homeostasis to counteract necrosis [52]; (iv) Autophagy modulation—NUPR1 promotes lysosomal biogenesis through Transcription factor binding to IGHM enhancer 3 (TFE3) activation and facilitates autophagosome–lysosome fusion by upregulating Synaptosomal-associated protein 25 (SNAP25) [25, 69]; (v) Therapy resistance—NUPR1 regulates p21 localization [2, 13, 44, 93], activates Erb-b2 receptor tyrosine kinase 2 (ERBB2) signaling, and maintains protective autophagy, thereby conferring multidrug resistance across treatment modalities [51]. Given its pleiotropic roles in multiple oncogenic processes, NUPR1 has emerged as a highly promising target for anticancer drug development.

Recent advances in NUPR1-targeted drug discovery have yielded promising early results. The small-molecule inhibitor ZZW-115 binds NUPR1 with high affinity and disrupts its transcriptional activity [49, 79]. In vitro and in vivo studies demonstrate that ZZW-115 suppresses NUPR1-mediated stress adaptation, thereby promoting apoptosis and ferroptosis while reducing tumor cell tolerance to metabolic and chemotherapeutic stress [42]. Moreover, ZZW-115 perturbs NUPR1-dependent autophagy and energy metabolism, thereby impairing tumor cell survival and adaptability through multiple mechanisms [42, 49, 79]. Additional evidence indicates that ZZW-115 may enhance antitumor immunity by remodeling the tumor immune microenvironment [66]. Combination therapy with ZZW-115 and conventional agents such as sorafenib has demonstrated significant efficacy in preclinical models of pancreatic and hepatocellular carcinoma (HCC) [50]. Although current evidence remains largely preclinical, the discovery of ZZW-115 provides a crucial experimental foundation for NUPR1-targeted anticancer strategies and opens new avenues for precision oncology.

This review systematically summarizes NUPR1 expression patterns across malignancies and their correlation with clinical outcomes, emphasizing its multidimensional regulatory networks during tumor initiation and progression—including microenvironmental modulation, metabolic reprogramming, regulation of apoptosis and ferroptosis, autophagy activation, and therapy resistance. Notably, crosstalk between NUPR1 and the tumor immune microenvironment represents an emerging research frontier. Its potential roles in modulating immune-cell infiltration and establishing immunosuppressive niches warrant further investigation, which may provide novel insights into mechanisms of immunotherapy resistance and combination strategies. Structure-based drug-design approaches have led to the development of targeted inhibitors, such as ZZW-115 and AJO14, which exhibit promising potential in precision oncology [64]. This review integrates basic and translational findings to construct a multidimensional framework for understanding NUPR1 biology and to propose innovative therapeutic strategies for the diagnosis and treatment of malignant tumors and related disorders.

## Expression in cancer and clinical prognosis

In recent years, NUPR1 has garnered considerable attention as a key regulator in tumor biology. Beyond its central role in orchestrating cellular responses to diverse stressors—including endoplasmic reticulum and metabolic stress—NUPR1 functions as a non-mutational stress-adaptation hub with oncogenic consequences that promotes tumor initiation, progression, metastasis, and therapeutic resistance across multiple malignancies. Aberrant NUPR1 expression is tightly associated with malignant phenotypes, including uncontrolled proliferation, resistance to apoptosis, enhanced invasion and metastasis, and extensive remodeling of the tumor microenvironment [6, 26, 100, 114]. Deciphering the intricate regulatory network of NUPR1 and its stage-specific functions in cancer progression is crucial for understanding tumor evolution and represents a promising avenue for prognostic assessment and targeted therapeutic intervention. Multiple studies have demonstrated that NUPR1 is markedly overexpressed in a broad spectrum of malignancies [88, 100]. Analyses of The Cancer Genome Atlas (TCGA) and Human Protein Atlas (HPA) datasets reveal consistently elevated NUPR1 expression across more than ten cancer types—including pancreatic ductal adenocarcinoma (PDAC), HCC, breast cancer (BC), colorectal cancer (CRC), and non-small cell lung cancer (NSCLC)—with tumor tissues showing significantly higher expression than adjacent normal counterparts. In pancreatic cancer, elevated NUPR1 expression accelerates the progression of pancreatic intraepithelial neoplasia (PanIN) and correlates strongly with increased metastatic potential and reduced overall survival [10, 34, 80]. In pancreatic tumorigenesis, NUPR1, RelB, and IER3 were shown to be co-expressed at the protein level in Kras^G12D^-driven PanIN lesions, and immunostaining of human PDAC specimens further supports co-activation of this axis in tumors, and co-overexpression of NUPR1 with RelB and IER3 frequently associates with higher recurrence rates and diminished responsiveness to chemotherapy [10, 34, 80]. In patients with early-stage breast cancer (Phase I/II Clinical Trial), elevated NUPR1 levels are significantly associated with recurrence and distant metastasis [75], leading to markedly shorter relapse-free and overall survival and conferring a higher likelihood of tamoxifen resistance in treated patients [95]. A similar pattern is observed in HCC, where NUPR1 expression is markedly upregulated in tumor tissues [2, 7, 100, 114]. High NUPR1 expression closely correlates with poorer overall and recurrence-free survival and is directly linked to sorafenib resistance, identifying it as an important molecular marker of unfavorable therapeutic outcomes [50]. Moreover, aberrantly high NUPR1 expression in colorectal cancer, NSCLC and other malignancies parallels reduced patient survival and increased recurrence risk [69, 106]. Pan-cancer analyses further confirm that elevated NUPR1 expression is almost invariably associated with shorter survival, increased metastatic potential, and enhanced therapeutic resistance, underscoring its utility as a cross-cancer prognostic biomarker. Although its biological functions encompass diverse signaling pathways—including stress adaptation, autophagy maintenance, metabolic reprogramming, and cell-death resistance—the most striking clinical observation is that persistent NUPR1 overexpression independently serves as a robust predictor of poor prognosis across cancers (Table 1).

**Table 1:** Studies of NUPR1 in clinical cancers

Pathological condition	Core signaling pathway	Experimental model	Intervention	Mechanism–phenotype description	Functional classification	References
Breast cancer	NUPR1/TEV/CH25H/IFNAR1/IFN1	Cells and breast cancer mouse model	Gene manipulation	NUPR1 enhances the TEV–CH25H–IFN1 signaling, activates the IFNAR1 pathway, and promotes distant metastasis and lung nodule formation	Metastasis enhancement	[71]
	NUPR1/ESR1	Cells and subcutaneous xenograft mouse model	Gene manipulation	NUPR1 upregulates ESR1 and cooperatively regulates autophagy-related genes such as BECN1, maintaining autophagic flux and promoting proliferation and tumor growth	Autophagy regulation	[95]
	NUPR1/TFE3	Cells, subcutaneous xenografts in nude mice, and clinical samples	Gene manipulation	NUPR1 activates TFE3 to promote lysosomal biogenesis, sustain autophagic function, and enhance tumor proliferation and invasion	Autophagy regulation	[105]
Pancreatic cancer	NUPR1/RelB/IER3	Cells, Pdx1-cre;LSL-Kras^G12D^ mice, and clinical tissue microarray	Gene manipulation	NUPR1 drives the RelB–IER3 axis to inhibit caspase-3–dependent apoptosis and promote pancreatic intraepithelial neoplasia formation	Apoptosis resistance	[34]
	NUPR1/AURKA	Human pancreatic cancer cell lines	MLN8237	NUPR1 activates AURKA, promotes DNA repair, and suppresses stress-induced autophagic cell death, thereby enhancing cell survival	Metabolic regulation/apoptosis resistance	[35]
	NUPR1/IER3/Caspase3, NUPR1/TGFβ/SMAD4/N-cadherin/β-catenin	Transgenic mice and primary cells	MLN8237	NUPR1 inhibits apoptosis through IER3 and cooperates with the TGFβ–SMAD4 axis to promote EMT, driving pancreatic cancer progression	Apoptosis resistance/EMT transition	[9]
Hepatocellular carcinoma	NUPR1/RELB/IER3/RUNX2	Multiple human hepatocellular carcinoma cell lines, subcutaneous tumors in mice, and patient samples	MLN8237	The NUPR1–RELB–IER3–RUNX2 signaling axis decreases sorafenib sensitivity in HCC, enhancing drug resistance and tumor growth	Chemoresistance/apoptosis resistance	[23]
	NUPR1/GRN	Human HCC cell lines, mitochondrial inhibition model, Rho0 cells, and patient samples	MLN8237	NUPR1 activates GRN and regulates the PI3K/AKT pathway to maintain glycolysis and mitochondrial function, thereby increasing invasiveness	Metabolic regulation/invasion enhancement	[52]
	NUPR1/ERK/JNK, NUPR1/ARG1/TREM2/VEGFB	THP1 cells, murine BMDMs, HCC samples, and xenograft tumors	TFP-2HCL, ZZW-115-2HCL	NUPR1 promotes TAM polarization, suppresses immune checkpoints, induces T-cell exhaustion, and reduces ICB sensitivity	Immune evasion/ICB resistance	[7]
	NUPR1/SREBP1/FASN/SCD1/FADS1/FADS2	Cell lines, xenograft tumors in nude mice, and patient tissues	Fatostatin, C75	NUPR1 facilitates mSREBP1 nuclear translocation, drives lipogenic enzyme expression and lipid droplet formation, and enhances tumor invasiveness and drug response modulation	Metabolic regulation/invasion enhancement	[100]
Lung cancer	NUPR1/IRE1/XBP1/VEGFANUPR1/PERK/eIF2α/ATF4/VEGFA	Human lung cancer cell lines and HUVECs	Gene manipulation	NUPR1 regulates the UPR–VEGF pathway to promote angiogenesis, migration, and expression of key effector molecules	Metabolic regulation/angiogenesis	[96]
	NUPR1/SNAP25/VAMP8	NSCLC cell lines, patient tissues, and SCID mouse xenografts	Gene manipulation	NUPR1 promotes formation of the SNAP25–VAMP8–SNARE complex, enhances autophagosome–lysosome fusion, and prevents premature senescence	Autophagy regulation	[69]
	NUPR1/LCN2	KP spontaneous mouse model, AT2 organoids, and human LUAD tissues	ZZW-115	NUPR1 upregulates LCN2, suppresses ferroptosis, maintains AT2 stemness, and regulates tumor initiation and progression	Ferroptosis resistance/stemness maintenance	[122]
Glioblastoma	NUPR1/LCN2	GBM cell lines	TFP	NUPR1 upregulates LCN2 to inhibit lipid peroxidation, enhance ferroptosis tolerance, and promote radio/chemotherapy resistance	Ferroptosis resistance/therapy resistance	[85]
Multiple myeloma	NUPR1/PI3K/AKT/mTOR	Multiple myeloma cell lines and bone marrow samples	LY294002	NUPR1 regulates autophagy-related genes; activation of the PI3K/AKT/mTOR pathway enhances autophagy and anti-apoptotic activity while suppressing apoptosis	Autophagy regulation/apoptosis resistance	[54]
Oral squamous cell carcinoma	NUPR1/TFE3	OSCC tissue microarray, cell lines, and nude mouse model	RAPA, CQ	The NUPR1–TFE3 axis activates lysosomal biogenesis, enhances autophagic flux, and promotes proliferation and metastasis	Autophagy regulation/metastasis enhancement	[25]
Ovarian cancer	NUPR1/AKT	Human ovarian cancer cell lines and nude mouse xenografts	LY294002	NUPR1 activates AKT signaling to promote proliferation, inhibit apoptosis, enhance migration, and stimulate tumor growth	Metabolic regulation/apoptosis resistance	[111]
Diffuse large B-cell lymphoma	NUPR1/CHOP/TRIB3	GEO and TCGA cohorts, and DLBCL cell lines	LY294002	The NUPR1–CHOP–TRIB3 signaling axis is associated with prognostic subtypes and immune infiltration, regulating gene expression	Immune regulation/prognosis	[115]
Bladder cancer	NUPR1/PI3K/AKT, NUPR1/M2 macrophage	BLCA cell lines, macrophage co-culture, and database analysis	Erastin	NUPR1 activates the PI3K/AKT/LCN2 axis to suppress ferroptosis, promote M2 macrophage polarization, and facilitate immune evasion	Ferroptosis resistance/immune evasion	[117]
Cervical cancer	NUPR1/TFE3/PI3K/AKT	TCGA data, HeLa/SiHa cell lines, and nude mouse xenografts	3-MA	The NUPR1–TFE3–PI3K/AKT pathway regulates autophagy and migration while inhibiting apoptosis and promoting tumor growth	Autophagy regulation/migration/apoptosis resistance	[99]
Prostate cancer	NUPR1/AKT	Prostate cancer cell lines, nude mouse xenografts, and patient tissues	LY294002	NUPR1 activates AKT to enhance proliferation, confer anti-apoptotic capacity, and promote docetaxel resistance	Metabolic regulation/drug resistance	[94]

NUPR1, Nuclear protein 1; TEV, Tumor-derived extracellular vesicle; CH25H, Cholesterol 25-hydroxylase; IFNAR1, Interferon alpha/beta receptor 1; IFN1, Type I interferon; ESR1, Estrogen receptor 1; TFE3, Transcription factor E3; Pdx1-Cre, Pancreatic and duodenal homeobox 1-Cre; LSL-Kras^G12D^, Lox-Stop-Lox Kras^G12D^ knock-in; AURKA, Aurora kinase A; MLN8237, Alisertib (AURKA inhibitor); IER3, Immediate early response 3; TGF-β, Transforming growth factor beta; SMAD4, Mothers against decapentaplegic homolog 4; RELB, Reticuloendotheliosis viral oncogene homolog B; RUNX2, Runt-related transcription factor 2; GRN, Granulin; PI3K, Phosphoinositide 3-kinase; AKT, Protein kinase B; Rho⁰, Mitochondrial DNA-depleted (rho-zero) cells; ERK, Extracellular signal-regulated kinase; JNK, c-Jun N-terminal kinase; ARG1, Arginase 1; TREM2, Triggering receptor expressed on myeloid cells 2; VEGFB, Vascular endothelial growth factor B; THP-1, Human monocytic leukemia cell line THP-1; BMDMs, Bone marrow-derived macrophages; TFP-2HCl, Trifluoperazine dihydrochloride; ZZW-115-2HCl, ZZW-115 dihydrochloride; SREBP1, Sterol regulatory element–binding protein 1; FASN, Fatty acid synthase; SCD1, Stearoyl-CoA desaturase 1; FADS1, Fatty acid desaturase 1; FADS2, Fatty acid desaturase 2; IRE1, Inositol-requiring enzyme 1; XBP1, X-box–binding protein 1; VEGFA, Vascular endothelial growth factor A; PERK, Protein kinase RNA-like ER kinase; eIF2α, Eukaryotic initiation factor 2 alpha; ATF4, Activating transcription factor 4; HUVECs, Human umbilical vein endothelial cells; SNAP25, Synaptosomal-associated protein 25; VAMP8, Vesicle-associated membrane protein 8; SNARE, Soluble NSF attachment protein receptor; NSCLC, Non-small cell lung cancer; SCID, Severe combined immunodeficiency; LCN2, Lipocalin-2; KP, Kras/Trp53 (KP) spontaneous lung adenocarcinoma mouse model; AT2, Alveolar type II cell; LUAD, Lung adenocarcinoma; GBM, Glioblastoma; mTOR, mechanistic target of rapamycin; LY294002, PI3K inhibitor LY294002; OSCC, Oral squamous cell carcinoma; RAPA, Rapamycin; CQ, Chloroquine; GEO, Gene Expression Omnibus; TCGA, The Cancer Genome Atlas; DLBCL, Diffuse large B-cell lymphoma; BLCA, Bladder cancer; 3-MA, 3-methyladenine; ICB, Immune checkpoint blockade

## The role of NUPR1 in tumor cell stress adaptation and homeostasis maintenance

**Fig. 1:**
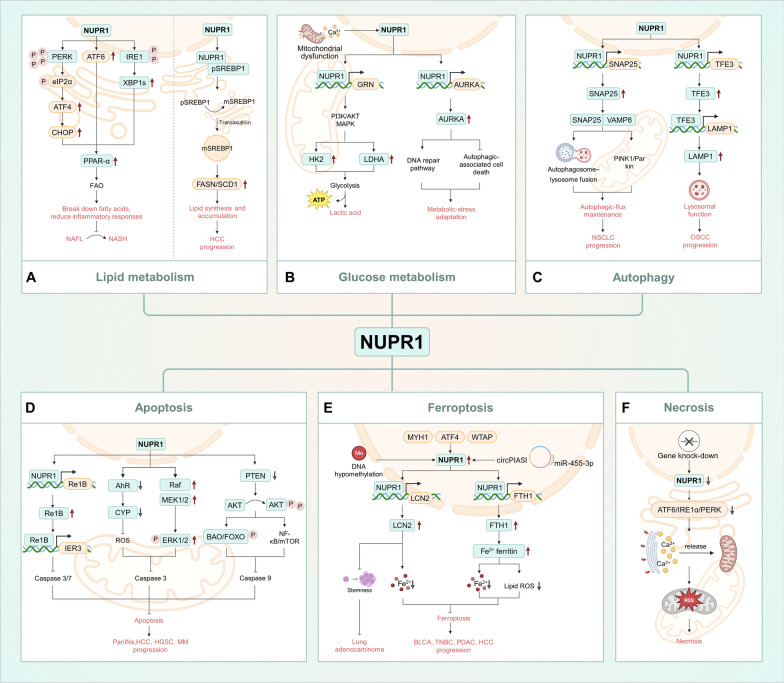
NUPR1 as a central node in cellular stress responses. **A** Lipid Metabolic Reprogramming: Stress-induced activation of the UPR stimulates PPARα to promote fatty acid oxidation and mitigate lipotoxicity; in HCC, the NUPR1–SCAP–SREBP1 axis enhances de novo lipogenesis. **B** Glucose Metabolic Reprogramming: Mitochondrial stress-induced calcium overload upregulates NUPR1, which enhances glycolysis through the PI3K–AKT/MAPK pathways; under glucose deprivation, the NUPR1–AURKA axis promotes DNA repair and suppresses autophagy-associated cell death. **C** Autophagic Flux and Lysosomal Homeostasis: The NUPR1–TFE3 axis promotes lysosome biogenesis and acidification; the NUPR1–SNAP25–VAMP8 complex enhances autophagosome–lysosome fusion and cooperates with the PINK1/Parkin pathway to maintain mitophagy. **D** Anti-Apoptosis: The NUPR1–RelB–IER3 axis inhibits mitochondrial apoptosis; the NUPR1–AhR–CYP pathway reduces ROS accumulation and suppresses apoptosis; in some solid tumors, NUPR1 inhibits apoptosis through the AKT signaling pathway. **E** Anti-Ferroptosis: The circPIAS1–miR-455-3p–NUPR1–FTH1 and NUPR1–LCN2 axes reduce free Fe^2^⁺ levels and lipid peroxidation, conferring ferroptosis resistance to tumor cells; in aged AT2 cells, the NUPR1–LCN2 axis suppresses stemness and delays tumor initiation. **F** Anti-Necrosis: NUPR1 maintains UPR activity, Ca^2^⁺/mitochondrial homeostasis, and OXPHOS to prevent energy crisis–induced and membrane rupture-associated necrosis. In the figure, solid arrows denote activation/promotion, while T-bars denote inhibition/negative regulation. This figure is a schematic representation and not drawn to scale. It was created using BioRender based on integrated findings from multiple studies (see “The Role of NUPR1 in Tumor Cell Stress Adaptation and Homeostasis Maintenance” section and References). For specific data or statistical methods, please refer to the original research. *Abbreviations* NUPR1, nuclear protein 1; UPR, unfolded protein response; IRE1, inositol-requiring enzyme 1; XBP1s, spliced X-box binding protein 1; SCAP, SREBP cleavage-activating protein; SREBP1, sterol regulatory element-binding protein 1; PPARα, peroxisome proliferator-activated receptor alpha; LDHA, lactate dehydrogenase A; PI3K/AKT, phosphoinositide 3-kinase/protein kinase B; TFE3, transcription factor E3; SNAP25/SNARE/VAMP8, synaptosomal-associated protein 25/soluble NSF attachment protein receptor/vesicle-associated membrane protein 8; PINK1/Parkin, PTEN-induced kinase 1/Parkin (E3 ubiquitin ligase); LCN2, lipocalin-2; FTH1, ferritin heavy chain 1; AhR, aryl hydrocarbon receptor; CYP, cytochrome P450; ROS, reactive oxygen species; BAX/BAK, BCL-2-associated X protein/BCL-2 antagonist/killer; OXPHOS, oxidative phosphorylation; BC, breast cancer; BLCA, bladder cancer; GBM, glioblastoma (multiforme); HCC, hepatocellular carcinoma; LUAD, lung adenocarcinoma; MM, multiple myeloma; NSCLC, non-small cell lung cancer; OC, ovarian cancer; OSCC, oral squamous cell carcinoma; PDAC, pancreatic ductal adenocarcinoma; TNBC, triple-negative breast cancer

As a central node in the cellular stress-response network, NUPR1 integrates metabolic reprogramming, autophagy control, and multiple cell-death pathways to reallocate energy and biosynthetic resources, thereby sustaining the remarkable adaptive resilience of tumor cells within hostile microenvironments (Fig. 1).

### Metabolic reprogramming

Metabolic reprogramming is a defining hallmark of cancer, characterized by the restructuring of metabolic networks to maintain a dynamic balance between energy supply and biosynthetic demand [97, 98]. Unlike normal cells that depend primarily on mitochondrial oxidative phosphorylation, cancer cells preferentially engage aerobic glycolysis—the Warburg effect—even in the presence of oxygen. Although this metabolic shift reduces ATP-generation efficiency, it rapidly produces carbon skeletons and reducing equivalents required for anabolic biosynthesis. In addition, cancer cells activate alternative routes—including glutaminolysis and fatty-acid oxidation—to build metabolic reserves, thereby enhancing adaptability to hypoxia, acidosis, and other microenvironmental stresses [56]. Such metabolic reconfiguration not only fulfills the proliferative requirements of tumor cells but also reshapes the tumor microenvironment via intermediates such as lactate, which suppress immune effector functions and facilitate immune evasion—forming a fundamental basis for tumor progression and therapeutic resistance [17, 38, 57, 101].

#### Lipid metabolic reprogramming

Within lipid-metabolism regulation, NUPR1 has been identified as a pivotal hub connecting metabolic stress with hepatocarcinogenesis [89, 112]. In clinical cohorts of morbidly obese patients with NAFLD/NASH, hepatic NUPR1 expression is dysregulated and varies with disease severity [89]. NUPR1 expression is higher in non-alcoholic fatty liver (NAFL) than in non-alcoholic steatohepatitis (NASH) and correlates inversely with the severity of hepatic steatosis. Patients with elevated NUPR1 levels show reduced hepatic-injury markers, suggesting a hepatoprotective role mediated through lipid-metabolism regulation [89]. Under high-fat-diet conditions, endoplasmic-reticulum stress in hepatocytes upregulates NUPR1, thereby activating unfolded-protein-response (UPR) pathways: (1) Protein kinase RNA-like ER kinase (PERK)-Activating transcription factor 4 (ATF4)-C/EBP homologous protein (CHOP), promoting adaptive translation and anti-apoptotic signaling; (2) Inositol-requiring enzyme 1 (IRE1)-Spliced X-box binding protein 1 (XBP1s), regulating lipid metabolism and counteracting lipotoxicity; and (3) Activating transcription factor 6 (ATF6), modulating lipid-metabolic gene expression [89]. Experimental evidence indicates that NUPR1-deficient mice develop more severe hepatic injury and increased lipid-droplet accumulation under a high-fat diet, underscoring NUPR1’s stress-protective function in non-tumorous states [89]. This metabolic-stress regulatory capacity becomes further amplified in tumors, where cancer cells exploit NUPR1-mediated adaptive networks to endure nutrient deprivation, oxidative stress, and therapeutic pressure, thereby gaining sustained survival advantages. Mechanistic studies reveal that NUPR1 enhances the nuclear translocation of the mature Sterol-Regulatory-Element-Binding Protein 1 (mSREBP1) by competitively binding the SCAP protein [100]. NUPR1 is specifically enriched at promoters of lipogenic genes such as Fatty acid synthase (*FASN)* and Stearoyl-CoA desaturase 1 (*SCD1)*, driving de novo lipogenesis and promoting HCC progression [100]. Notably, NUPR1 preserves lipid-raft integrity, thereby mitigating free-fatty-acid-induced toxicity. In pancreatic cancer cell models (in vitro), genetic inactivation/knockdown of NUPR1 reduces mitochondrial membrane potential, impairs mitochondrial/OXPHOS function, and exacerbates metabolic-stress-induced injury [77]. In summary, NUPR1 exerts dual roles in hepatic-lipid-metabolism regulation—both protective and tumor-promoting. Under non-tumorous conditions, NUPR1 mitigates lipotoxicity and protects hepatocytes; however, during hepatocarcinogenesis it promotes lipid synthesis and de novo lipogenesis, thereby accelerating tumor progression and demonstrating a context-dependent functional switch.

#### Glucose metabolic reprogramming

In glucose-metabolism regulation, NUPR1 has been identified as a key regulator of metabolic reprogramming triggered by mitochondrial dysfunction [52]. Comparative transcriptomic analyses across three models—HCC cells with intrinsic mitochondrial defects, respiration-chain-inhibitor-induced models, and mitochondrial-DNA-depleted Rho⁰ cells—identified NUPR1 as a commonly upregulated gene, underscoring its pivotal role in compensatory glycolysis after oxidative-phosphorylation impairment [52]. Mechanistic studies show that mitochondrial-dysfunction-induced calcium overload markedly activates NUPR1 transcription; treatment with the calcium ionophore A23187 significantly increases NUPR1 mRNA levels, whereas the calcium chelator BAPTA-AM completely abolishes this effect, underscoring the importance of calcium signaling in NUPR1 activation [52]. Further studies demonstrate that NUPR1 binds the promoter region (22,033–21,547 bp) of the *GRN* gene, activating PI3K/AKT and Mitogen-activated protein kinase (MAPK) pathways and thereby upregulating key glycolytic enzymes Hexokinase 2 (*HK2)* and Lactate dehydrogenase A (*LDHA*) [52]. Collectively, these alterations markedly increase glycolytic flux, redirecting glucose metabolism from oxidative phosphorylation toward lactate fermentation, which promotes rapid lactate accumulation and sustains ATP production—conferring continuous survival advantages under energy-limited conditions. Conversely, in human hepatoma cell lines in vitro, NUPR1 silencing significantly reduces glycolytic activity and lactate generation, impairing energy supply and disrupting metabolic homeostasis [52]. Overall, NUPR1 couples calcium signaling with metabolic-network remodeling to reshape the glycolytic phenotype of HCC cells, acting as a central hub for energy-adaptive reprogramming under mitochondrial dysfunction. Unlike the active promotion of glycolysis by NUPR1 in HCC, loss of NUPR1 in pancreatic cancer impairs mitochondrial respiratory chain complex IV activity and reduces OXPHOS capacity, thereby driving a secondary compensatory state of “glycolytic dependence”, which further highlights the central role of NUPR1 in regulating metabolic plasticity [34, 35, 52].

Beyond glycolytic compensation, NUPR1 also serves as a crucial regulatory node for cell survival under energy-deprivation and metabolic-stress conditions. Under metabolic-stress conditions such as glucose deprivation, NUPR1 expression is markedly upregulated, acting as a key determinant of tumor-cell survival [35, 36]. Studies show that NUPR1 activates the downstream Aurora-kinase A (AURKA) pathway, transcriptionally enhancing multiple DNA-repair and cell-cycle regulators—including *RAD51*, *BRCA1*, *CDC23*, *POLQ* and *ATR*—thereby preserving genomic stability and mitigating DNA-damage accumulation [35, 36]. Meanwhile, the NUPR1–AURKA axis suppresses metabolic-stress-induced autophagy-associated cell death—a predominantly caspase-independent process—underscoring its role in regulating non-canonical cell-death pathways [35, 36]. In human PDAC cell lines, loss-of-function assays confirm that NUPR1 silencing markedly reduces AURKA levels, rendering cells hypersensitive to metabolic stress, with increased DNA damage, elevated autophagic activity, and higher cell-death rates [35, 36]. Overall, NUPR1 establishes a metabolic-stress-adaptation pathway through AURKA-mediated promotion of DNA repair and suppression of autophagy, markedly enhancing tumor-cell survival under glucose deprivation and energy-limiting microenvironments. This mechanism highlights NUPR1 as a central component of the metabolic-stress-DNA-repair-cell fate network, positioning it as a molecular hub that enables tumor cells to withstand extreme metabolic stress and maintain survival advantages [36].

Current evidence indicates that NUPR1 drives tumor metabolic reprogramming through multilayered networks integrating lipid synthesis, glycolytic activation, and calcium-signaling control, providing a conceptual basis for developing NUPR1-targeted metabolic interventions. Future investigations should elucidate the interplay between NUPR1 and immunometabolism within the tumor microenvironment and define its dynamic regulatory mechanisms underlying metabolic heterogeneity and therapeutic resistance.

### NUPR1 as a central regulator of tumor cell autophagy

Autophagy is a highly conserved intracellular degradation process in which double-membrane autophagosomes sequester misfolded proteins and damaged organelles, subsequently fusing with lysosomes where hydrolytic enzymes degrade the cargo [47, 86]. This process is orchestrated by autophagy-related gene (ATG) products and ultimately releases metabolites that support biosynthesis and energy metabolism. Beyond fueling cells under nutrient deprivation, autophagy is central to stress adaptation and homeostasis by clearing toxic protein aggregates and maintaining organelle quality control. During tumor initiation and progression, autophagy is dual-edged: it can suppress malignant transformation by limiting oxidative damage, yet it also supports tumor proliferation and microenvironmental adaptation by supplying metabolic intermediates [27, 53, 68, 102].

In oral squamous cell carcinoma (OSCC), NUPR1 directly activates TFE3, driving the expression of lysosome-biogenesis genes [26]. NUPR1 suppression reduces TFE3 levels, lowers Lysosomal-associated membrane protein 1 (LAMP1) expression, diminishes lysosomal proteolysis and acidification, and ultimately blocks autophagic flux. Notably, NUPR1 deficiency primarily impairs lysosomal function without disrupting autophagosome formation or autophagosome–lysosome fusion [26]. Exogenous TFE3 rescues lysosomal activity and reduces accumulation of Microtubule-associated protein 1A/1B-light chain 3 beta-II (LC3B-II) and Sequestosome 1 (p62) [26], confirming that the NUPR1–TFE3 axis promotes tumor invasion by maintaining lysosomal homeostasis. A similar mechanism operates in cervical and breast cancers, indicating a broadly pro-tumorigenic pathway [25]. In non-small cell lung cancer (NSCLC), NUPR1 upregulates the synaptic-associated protein SNAP25, which complexes with Vesicle-associated membrane protein 8 (VAMP8) to form a Soluble NSF attachment protein receptor (SNARE) machinery mediating autophagosome–lysosome fusion [43, 69]. NUPR1 silencing induces autophagosome accumulation, impairs mitophagy, and precipitates a premature-senescence phenotype. SNAP25 cooperates with the PTEN-induced kinase 1 (PINK1)/E3 ubiquitin ligase Parkin pathway to enhance mitophagy, thereby maintaining metabolic homeostasis and promoting tumor progression [69].

NUPR1 function is strongly context-dependent within specific signaling networks. In OSCC, NUPR1 counteracts PI3K/AKT/Mechanistic target of rapamycin (mTOR) signaling to prevent cytoplasmic retention of TFE3, reversing the pathway’s inhibitory effect on lysosome biogenesis; in NSCLC, the NUPR1–SNAP25 axis appears independent of canonical autophagy regulators and directly enhances autophagosome maturation. This regulatory heterogeneity suggests that NUPR1 integrates cell-type-specific nodes to adaptively tune autophagic flux across cancers, ultimately conferring survival advantages.

### NUPR1-mediated cell death regulatory mechanisms

Cell death is a fundamental process that maintains tissue homeostasis and shapes disease progression. The principal modalities include apoptosis, ferroptosis, and necrosis. These modalities are tightly interconnected under physiological and pathological conditions, collectively influencing tumor initiation, progression, and therapeutic response [8, 19, 32, 48]. Recent studies identify NUPR1—a stress-response regulator—as a pivotal modulator of multiple cell-death pathways [34, 61, 77]. NUPR1 modulates apoptosis via pathways involving the B-cell lymphoma 2 (BCL-2) family and p53 and suppresses ferroptosis by upregulating ferroptosis-inhibitory mediators [13, 61]. Moreover, NUPR1 cross-regulates intracellular oxidative-stress, autophagy, and necroptosis pathways. Aberrant activation or overexpression of NUPR1 confers resistance to multiple death signals, thereby promoting tumorigenesis, therapy resistance, and disease progression. Elucidating how NUPR1 regulates cell death is critical for understanding immune evasion and therapy resistance within the tumor microenvironment and may inform development of anticancer strategies targeting death pathways.

#### Regulation of apoptosis

Within highly stressed tumor microenvironments, cancer cells remodel molecular networks to resist death signals, maintain metabolic homeostasis, and sustain growth. As a key stress-response molecule, NUPR1 is robustly activated in poorly vascularized, nutrient-deprived microenvironments and mediates anti-apoptotic protection through cascaded mechanisms [80]. Across cancers, NUPR1 exhibits tumor-specific regulatory patterns. In the *Kras*^*G12D*^-driven PanIN model, NUPR1 knockout completely prevents PanIN formation [9, 30, 31]. NUPR1 directly drives *RelB* transcription by binding its promoter as a chromatin-associated factor, thereby promoting transcriptional activation. In the absence of NUPR1, *RelB* mRNA and protein are not adequately induced under stress. RelB—a key transcription factor of the noncanonical NF-κB pathway—activates the downstream anti-apoptotic factor *IER3* [1, 34]. NUPR1 upregulation increases *RelB*, which in turn induces *IER3*. *IER3* induction depends on the NUPR1–RelB axis rather than the canonical RelA/p65 pathway. Deletion of either *RelB* or *IER3* sensitizes cells to stress-induced death, with reduced viability and increased caspase-3/7 activation [34]. IER3 inhibits mitochondrial apoptosis by suppressing caspase-3/7 activation and blocking Bax/Bak-mediated signaling. IER3 knockdown abolishes the anti-apoptotic effect of the NUPR1–RelB axis, indicating an indispensable NUPR1–RelB–IER3 cascade. IER3 both suppresses caspase activation and negatively regulates RelA/p65, providing feedback control over NF-κB signaling. In *Kras*^*G12D*^ GEMMs, pancreas-specific *RelB* deletion delays PanIN development and abolishes *IER3* expression, confirming the essential role of the NUPR1–RelB–IER3 axis in early tumorigenesis [34, 35, 52]. In HCC, NUPR1 interacts with the aryl hydrocarbon receptor (AhR), promotes its autophagic degradation, reduces AhR nuclear translocation and transcriptional activity [114], suppresses downstream cytochrome P450 (CYP) expression, limits excessive Reactive oxygen species (ROS), decreases caspase-3 activation, and reduces apoptosis. ROS scavengers or CYP inhibitors reverse the apoptosis induced by NUPR1 loss in HCC cells, confirming that the AhR/CYP/ROS axis mediates its anti-apoptotic effect [114]. In glioblastoma, elevated NUPR1 enhances Extracellular signal-regulated kinase (ERK1/2) and p38 phosphorylation, downregulates caspase-3, and inhibits apoptosis [85]. These findings suggest that NUPR1 regulates cell survival via MAPK signaling. In ovarian cancer, NUPR1 silencing downregulates BCL-2 and BCL-XL, reduces Cyclin-dependent kinase 4/6 (CDK4/6), and increases pro-apoptotic proteins (caspase-3, caspase-9, Bax), an effect attributable to AKT-pathway suppression [111]. The role of NUPR1 is more extensively characterized in multiple myeloma. NUPR1 and autophagy-related genes (*BECN1*, *ATG5*, *ATG12*) are upregulated in myeloma cells, and NUPR1 expression correlates positively with clinical stage, serum calcium, and bone-marrow plasma-cell proportion [54]. Upon NUPR1 silencing, PI3K/AKT/mTOR signaling shifts: phosphorylated PI3K/AKT/mTOR increase, ATG5/*BECN1* and LC3-II/LC3-I decrease, and p62 accumulates—collectively indicating reduced autophagy and enhanced apoptosis. Notably, rapamycin partially rescues apoptosis caused by NUPR1 silencing, whereas 3-methyladenine (3-MA) amplifies it, indicating that NUPR1 indirectly regulates apoptosis through autophagy modulation [54]. Through multilayered networks, NUPR1 synergistically suppresses tumor-cell apoptosis, enabling survival advantages under stress. This function appears both conserved and tissue-specific across tumors, offering insights into mechanisms of tumor tolerance and progression.

#### Regulation of ferroptosis

As a regulator of iron metabolism, NUPR1 exhibits pronounced cell-type dependence. NUPR1 suppresses ferroptosis primarily via its downstream effector lipocalin-2 (LCN2), inhibiting lipid peroxidation and protecting cells. In pancreatic cancer, ferroptosis inhibitors induce ER stress and activate ATF4, which upregulates NUPR1. NUPR1 then binds the *LCN2* promoter, promotes iron-efflux programs, reduces intracellular Fe^2^⁺, and suppresses Fenton-driven lipid peroxidation—thereby conferring ferroptosis resistance via the LCN2 axis [61]. Similarly, in bladder cancer, NUPR1 is activated by Myosin heavy chain 11 (*MYH11*) and inhibits ferroptosis through the PI3K/AKT/LCN2 axis [117]. In triple-negative breast cancer (TNBC), Wilms tumor 1-associating protein (*WTAP*) activation of NUPR1 upregulates *LCN2*. NUPR1 silencing increases intracellular Fe^2^⁺, lowers the GSH/GSSG ratio, enhances lipid peroxidation, upregulates *ACSL4*, downregulates *GPX4*, and induces ferroptosis [87]. In glioblastoma, cerebrospinal-fluid-derived stimuli upregulate NUPR1, transcriptionally activate *LCN2*, and confer resistance to radio- and chemotherapy [85]. In HCC, NUPR1 operates within the circPIAS1/miR-455-3p/NUPR1 network to transcriptionally activate *FTH1*, promoting Fe^2^⁺ sequestration in ferritin cores and reducing the labile iron pool [119].

Because ferroptosis is an iron-dependent, ROS-driven programmed cell death [3, 19, 84], NUPR1 activation under oxidative stress—and its role in sustaining redox homeostasis and mitochondrial integrity—is particularly critical. Mechanistically, ROS accumulation induces NUPR1 nuclear translocation. In the nucleus, NUPR1 binds AhR, blocks AhR/ARNT nuclear translocation, and accelerates AhR degradation via lysosomal pathways [114]. Inhibiting AhR signaling downregulates cytochrome P450 (CYP) transcription and reduces electron-transport-chain-derived superoxide [114].

However, in aged LUAD-origin alveolar type II (AT2) cells, DNA-hypomethylation-driven NUPR1 overexpression hyperactivates *LCN2*, causing functional iron deficiency and ferroptosis resistance while suppressing stemness and delaying tumor initiation [122]. This highlights the spatiotemporal specificity of NUPR1-mediated iron regulation and suggests that therapeutic value must be evaluated within defined pathological contexts.

#### Regulation of necrosis

NUPR1 effectively protects against necrotic cell death. Iovanna’s group used siRNA and CRISPR–Cas9 to suppress NUPR1 in pancreatic cancer cells and observed marked necrotic features under basal conditions and following ER-stress induction. NUPR1 deficiency attenuates cellular ER-stress responsiveness, downregulating ATF6, IRE1α, and PERK and impairing the unfolded-protein response (UPR). Consequently, NUPR1-deficient in human pancreatic cancer cell lines show extensive ER Ca^2^⁺ release, mitochondrial Ca^2^⁺ accumulation, loss of mitochondrial-membrane potential, mitochondrial dysfunction, and increased ROS [77]. Mitochondrial oxidative phosphorylation (OXPHOS) declines, ATP synthesis falls sharply, and cellular energy supply is compromised. Although cells attempt compensation via enhanced glycolysis, its low efficiency depletes glucose rapidly and aggravates necrosis. In NUPR1-knockout mice with induced pancreatitis, serum LDH and lipase increase and pancreatic necrotic area/scores are elevated, indicating that NUPR1 protects acinar cells from necrotic injury. Necrostatin-1, an inhibitor of programmed necrosis, reverses necrosis induced by NUPR1 loss, whereas apoptosis inhibitors have no effect, confirming that NUPR1 prevents programmed necrosis primarily via energy-metabolism and stress-response control [77].

Nerve injury-induced protein 1 (NINJ1) has been identified as a terminal effector of plasma-membrane rupture during necrosis [16, 46]; however, whether NUPR1 intersects with NINJ1 remains unknown, and the molecular basis of NUPR1-mediated protection from necrosis requires clarification.

Through integrated stress-response programs, NUPR1 provides multilayered protection encompassing metabolism, cell death, and autophagy, enabling survival in hostile microenvironments. NUPR1 also promotes phase separation via interactions with Ras GTPase-activating protein-binding protein 1 (G3BP1), promoting liquid–liquid phase separation (LLPS), facilitating stress-granule (SG), formation and enabling KRAS-mutant tumor cells to adapt to and resist stress [78, 108]. This further broadens the functional scope of NUPR1 in stress regulation. Although NUPR1’s critical roles are evident across cancers, its spatiotemporal specificity and the diversity of its regulatory networks require further investigation. Precisely targeting NUPR1 may provide new avenues to overcome therapy resistance and malignant progression.

## NUPR1 remodels the immune microenvironment

Tumors are not merely the result of uncontrolled cancer-cell proliferation; they constitute complex, dynamic “ecosystems”, collectively termed the tumor microenvironment (TME) [37]. The TME comprises tumor cells, immune cells, fibroblasts, endothelial, and other stromal cells, together with an abundant extracellular matrix (ECM) and diverse soluble factors. Remodeling of the tumor immune microenvironment (TIME) is a central mechanism underlying poor prognosis and therapeutic resistance—particularly resistance to immunotherapy [4, 45]. Aberrant expansion and redistribution of immunosuppressive populations—especially regulatory T cells (Tregs), myeloid-derived suppressor cells (MDSCs), and tumor-associated macrophages (TAMs)—are key drivers of this process. Among these, TAMs are particularly critical owing to their plasticity and multifaceted roles in reshaping the immune microenvironment. In most solid tumors, TAMs predominantly adopt an M2-polarized phenotype, secreting immunosuppressive cytokines and pro-angiogenic factors [70, 82]. These TAMs suppress CD8⁺ effector T cells, expand Tregs, promote angiogenesis, and remodel the ECM, collectively establishing a highly immunosuppressive milieu. M2-type TAMs also upregulate checkpoint molecules such as Programmed death-ligand 1 (PD-L1), inducing T-cell exhaustion and thereby dampening immunotherapy responses while promoting immune evasion and resistance [20, 70, 121].

Recent studies show that tumor cells actively drive M2 polarization via lactate, chemokines, and exosomal signaling [14, 73, 121], while modulating effectors such as NUPR1 to reinforce immunosuppression. Enrichment of M2-type TAMs correlates strongly with greater tumor aggressiveness, increased metastatic risk, and poor prognosis [107, 113, 120]. Targeting TAM polarization and functional reprogramming has emerged as a promising immunotherapeutic strategy: inhibiting M2 polarization and promoting M1 transition can reshape the TIME, improving immunotherapy responsiveness and patient outcomes.

In HCC, NUPR1 is highly expressed in TAMs and associates closely with poor prognosis. NUPR1-high TAMs exhibit M2 polarization, secrete immunosuppressive cytokines, upregulate checkpoint molecules, induce CD8⁺ T-cell exhaustion, and orchestrate a strongly immunosuppressive microenvironment [7]. Mechanistic data indicate that high glycolytic activity in HCC cells generates lactylated histone H3K18 (H3K18la), which upregulates NUPR1 in TAMs [7]. NUPR1 suppresses downstream MAPK/ERK signaling, drives M1-to-M2 repolarization, and fosters a tumor-promoting, immunosuppressive milieu. Pharmacologic inhibition with TFP-2HCl or ZZW-115 reverses immunosuppression and enhances immunotherapy efficacy [7]. Combination therapy with PD-L1 blockade and ZZW-115 markedly alleviates TME immunosuppression and suppresses tumor growth [7]. Similarly, in bladder cancer, NUPR1 overexpression strongly drives TAM M2 polarization, whereas knockdown of the upstream regulator *MYH11* suppresses M2 polarization [117], although the precise mechanisms remain to be defined.

Notably, NUPR1 is not uniformly immunosuppressive within the TIME; rather, its function is strongly tumor-type- and context-dependent. In lung adenocarcinoma (LUAD), single-cell RNA-seq (scRNA-seq) and bulk RNA-seq identified M2-related and angiogenesis-associated programs, yielding a 12-gene prognostic model in which NUPR1 emerged as a core component [59]. Within this model, NUPR1 behaved as a protective factor: higher expression correlated with enriched immune-cell infiltration and a more activated antitumor immune microenvironment.

These observations suggest that NUPR1’s role is strongly shaped by cellular composition, metabolic state, and operative signaling networks within the TME. For example, in HCC and bladder cancer, high tumor glycolysis generates lactate that—via lactylation—upregulates NUPR1 in TAMs, driving M2 polarization and promoting an immunosuppressive, T-cell-exhausted microenvironment [7]. By contrast, in subsets of LUAD, high NUPR1 associates with greater immune infiltration and an activated antitumor phenotype, suggesting that, under specific molecular contexts, NUPR1 may enhance immune responsiveness [59]. NUPR1 is regulated by upstream signals such as MYH11/PI3K/AKT and, in turn, modulates downstream pathways including ferroptosis, macrophage polarization, and metabolic reprogramming. The functional orientation of NUPR1 is determined by tumor- and context-specific factors such as genetic alterations, molecular subtypes, and signaling activation states: For example, in bladder cancer, NUPR1 signals through the PI3K/AKT axis to suppress ferroptosis and promote M2 polarization, establishing a canonical immunosuppressive microenvironment [117]; in clear-cell renal cell carcinoma, NUPR1 inhibition induces ferroptosis and increases CD4⁺ and CD8⁺ T-cell infiltration [66]. In certain LUAD subgroups, NUPR1 correlates with active immune infiltration, potentially reflecting a compensatory activation or “reversal” within its regulatory network [59].

**Fig. 2:**
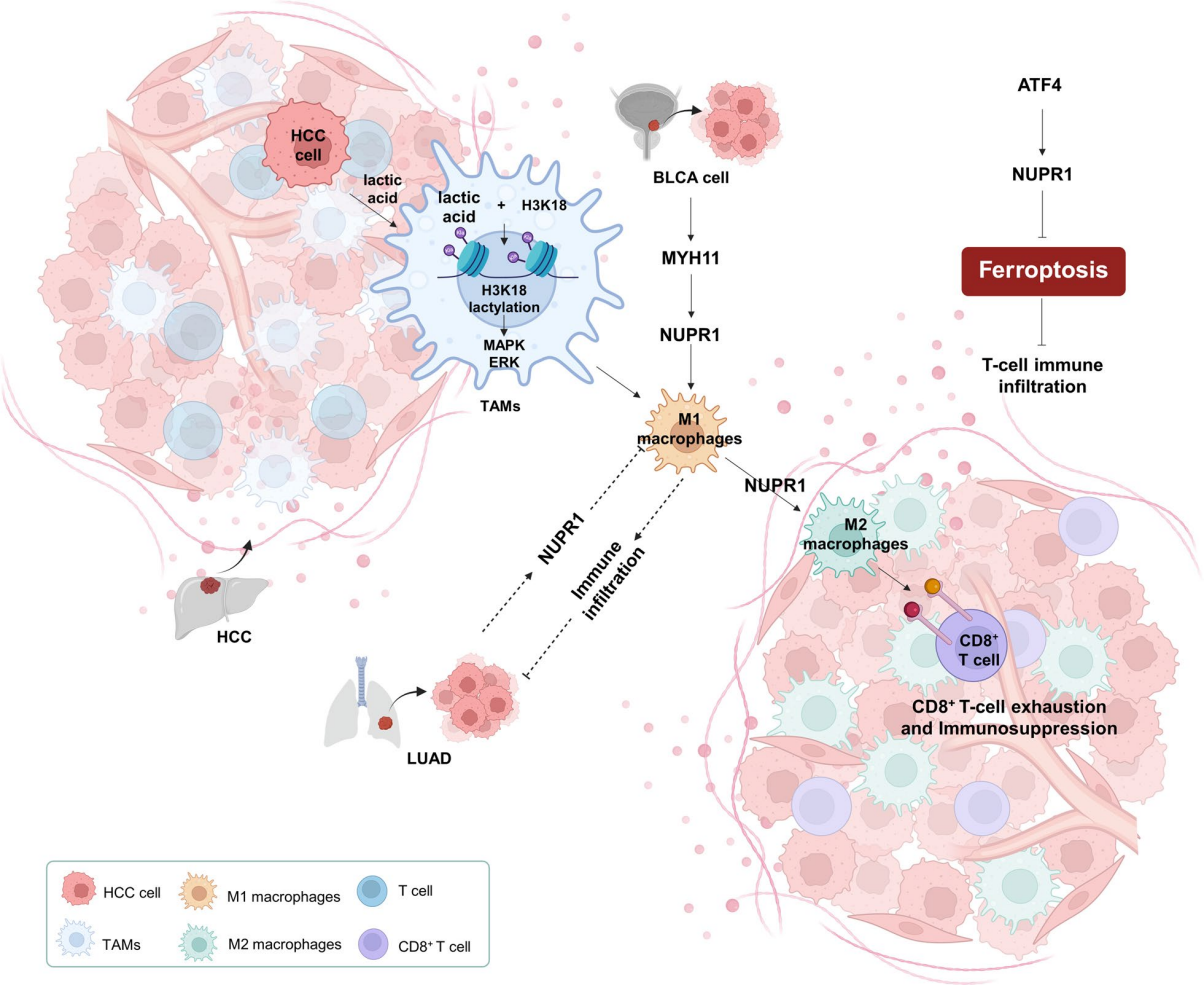
Context-dependent remodeling of the TME and TAM polarization driven by NUPR1. In HCC (Lactylation–NUPR1 axis): Tumor cells with high glycolytic activity generate lactate that induces H3K18 lactylation (H3K18la), leading to NUPR1 upregulation and MAPK/ERK inhibition, which drives M2 macrophage polarization and establishes an immunosuppressive microenvironment. In BLCA: The MYH11—PI3K/AKT—NUPR1 signaling cascade promotes M2 polarization and suppresses ferroptosis. In ccRCC: The ATF4–NUPR1 axis inhibits ferroptosis and reduces CD4⁺/CD8⁺ T-cell infiltration. In certain LUAD subgroups: High NUPR1 expression correlates with increased immune infiltration and potential protective immune responses. This figure is a schematic representation and not drawn to scale. It was created using BioRender based on integrated findings from multiple studies (see “NUPR1 Remodels the Immune Microenvironment” section and References). For specific data or statistical methods, please refer to the original research. *Abbreviations* TME, tumor microenvironment; TIME, tumor immune microenvironment; TAM(s), tumor-associated macrophage(s); Tregs, regulatory T cells; H3K18, histone H3 lysine 18; MAPK/ERK, mitogen-activated protein kinase/extracellular signal-regulated kinase

As a key node within the TME immune-regulatory network, NUPR1 exhibits highly context-dependent functions across cancer types. Its regulation is bidirectional and context-dependent, influenced by tumor type, microenvironmental composition, molecular background, and metabolic state. Defining its molecular mechanisms and immune-regulatory axes may pave the way for precision immunotherapy and resistance reversal, with significant implications for clinical stratification and personalized treatment (Fig. 2).

## NUPR1 as a multidimensional hub of therapeutic resistance

**Fig. 3:**
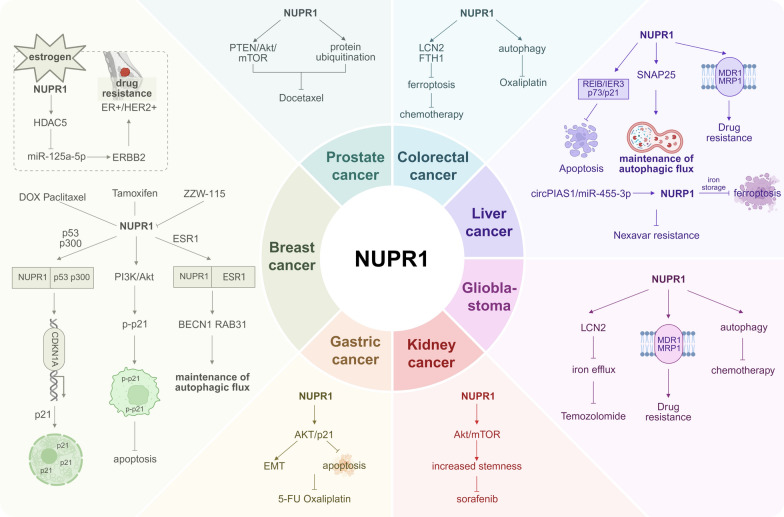
NUPR1 as a multidimensional hub of therapeutic resistance (pan-cancer overview). Breast cancer (BC): Chemotherapy (DOX/paclitaxel) induces stress-mediated NUPR1 upregulation; NUPR1 cooperates with p53/p300 to activate CDKN1A/p21, causing cell-cycle arrest; via PI3K–AKT signaling, promotes cytoplasmic p21 localization to inhibit TRAIL-induced apoptosis; with ESR1, co-regulates BECN1/RAB31 to sustain autophagic flux. In endocrine resistance, the NUPR1–HDAC5–miR-125a-5p–ERBB2 axis promotes HER2 upregulation; tamoxifen-induced NUPR1 increases resistance, which can be partially reversed by ZZW-115. Prostate Cancer (PCa): NUPR1 activates the PTEN/AKT/mTOR axis and ubiquitination networks, reducing sensitivity to docetaxel. Colorectal Cancer (CRC): NUPR1-dependent autophagy counteracts oxaliplatin-induced DNA damage; the FTO–NUPR1–LCN2/FTH1 axis enhances iron storage, suppresses lipid peroxidation, and reduces ferroptosis. Liver cancer—Hepatocellular Carcinoma (HCC): The NUPR1–p73–p21 and RELB–IER3–RUNX2 axes reduce sorafenib sensitivity; NUPR1 maintains autophagic flux via SNAP25 and upregulates MDR1/MRP1, contributing to multidrug resistance; the circPIAS1/miR-455-3p–NUPR1–FTH1 axis promotes iron storage and inhibits ferroptosis. Co-treatment with NSC59984 or ZZW-115 enhances sorafenib sensitivity. Glioblastoma (GBM): NUPR1 upregulates LCN2 (promoting iron efflux) and autophagy, while enhancing MDR1/MRP1 expression, conferring temozolomide resistance. Kidney cancer—Clear Cell Renal Cell Carcinoma (ccRCC): FTO-mediated NUPR1 upregulation activates the AKT/mTOR pathway and stemness programs (OCT4/SOX2/NANOG/CD44), enhancing sorafenib resistance. Gastric Cancer (GC): The YAP → NUPR1 → AKT/p21 axis promotes EMT and anti-apoptosis, increasing resistance to 5-FU and oxaliplatin. This figure is a schematic representation and not drawn to scale. It was created using BioRender based on integrated findings from multiple studies (see “NUPR1 as a Multidimensional Hub of Therapeutic Resistance” section and References). For specific data or statistical methods, please refer to the original research. *Abbreviations* PCa, prostate cancer; CRC, colorectal cancer; GC, gastric cancer; mTOR, mechanistic target of rapamycin; ESR1, estrogen receptor 1; BECN1, beclin 1; p21/CDKN1A, cyclin-dependent kinase inhibitor 1 A; ERBB2/HER2, erb-b2 receptor tyrosine kinase 2; MDR1/ABCB1, multidrug resistance protein 1 (P-glycoprotein); MRP1/ABCC1, multidrug resistance-associated protein 1; FTO, fat mass and obesity-associated protein; YAP, yes-associated protein; EMT, epithelial–mesenchymal transition; DOX, doxorubicin; 5-FU, 5-fluorouracil; Oxaliplatin; Paclitaxel; Docetaxel; Temozolomide; Sorafenib (Nexavar); Tamoxifen; NSC59984 (p73 agonist); ZZW-115 (NUPR1 inhibitor)

In routine clinical practice, patients with advanced cancers such as hepatocellular carcinoma, biliary tract cancer, and pancreatic cancer are commonly exposed to multiple lines of systemic therapy, including tyrosine-kinase inhibitors, anti-angiogenic agents, cytotoxic combinations, and immune-checkpoint blockade [55, 116]. However, comprehensive clinical data indicate that median survival remains limited and that disease progression due to primary or secondary resistance is nearly universal. Overcoming therapeutic resistance remains a central challenge in oncology. Resistance not only undermines the efficacy of chemotherapy, targeted therapy, and immunotherapy but also drives tumor recurrence and metastasis. Deciphering and reversing resistance mechanisms can directly improve response rates and survival, representing a key step toward precision oncology and durable disease control [29, 40, 65]. As a master regulator of tumor adaptation, NUPR1 centrally orchestrates multiple resistance mechanisms across cancer types. By dynamically modulating cell-death signaling and key stress-adaptation pathways, NUPR1 establishes a broad-spectrum resistance barrier across diverse cancers and treatment modalities (Fig. 3).

### Hepatocellular carcinoma

In HCC, NUPR1 levels are lower in sorafenib responders than in non-responders [2], suggesting utility as a predictive marker of resistance. NUPR1 silencing upregulates p73, which activates the cell-cycle inhibitor CDKN1A/p21 and increases sorafenib sensitivity [2]. Co-silencing NUPR1 and p73 abolishes this sensitization [2]. Combining the p73 activator NSC59984 with sorafenib yields synergy by disrupting NUPR1-mediated autophagic protection and promoting p73-dependent apoptosis, markedly enhancing sorafenib efficacy. The NUPR1–RELB–IER3–RUNX2 axis also contributes to sorafenib resistance in HCC cells [23]. Knockdown of NUPR1, RELB, IER3, or RUNX2 reprograms HCC cells toward less aggressive, less resistant phenotypes, providing mechanistic leverage for therapeutic reversal.

### Breast cancer

In triple-negative breast cancer (TNBC), microfluidic selection of doxorubicin-resistant cells (L-DOXR) followed by transcriptomics identified NUPR1 as the most upregulated resistance-associated gene [58], strongly correlating with poor prognosis. Elevated NUPR1 arises from HDAC11 downregulation, which hyperacetylates the NUPR1 promoter and relieves deacetylation-mediated repression [58]. ZZW-115 treatment, NUPR1 knockdown, or HDAC11 overexpression overcomes resistance and suppresses in-vivo tumor growth [58]. Under doxorubicin or paclitaxel stress, NUPR1 forms a complex with p53 and p300, binds the CDKN1A promoter, and drives nuclear p21 accumulation and Retinoblastoma protein (RB) phosphorylation, resulting in cell-cycle arrest [13]. Concurrently, NUPR1 activates PI3K/AKT signaling, inducing p21 phosphorylation and cytoplasmic translocation, which inhibits TRAIL-mediated apoptosis and enhances chemoresistance [13]. Inhibiting NUPR1 or p21 restores sensitivity to doxorubicin/paclitaxel and increases apoptosis [13]. During endocrine therapy, tamoxifen induces NUPR1, which cooperates with Estrogen receptor 1 (ESR1) to transcriptionally regulate autophagy genes such as *BECN1* and *RAB31*, maintaining autophagic flux and supporting survival [95]. NUPR1 loss raises lysosomal pH, disrupts autophagy, and induces premature senescence, whereas inhibition of the NUPR1–ESR1 axis dismantles the “autophagic shield” and restores tamoxifen sensitivity. Moreover, with prolonged endocrine therapy, some tumors transition from ER⁺ to HER2⁺ phenotypes with worse prognosis [51]. NUPR1 activates HDAC5, relieving miR-125a-5p-mediated repression of *ERBB2*, thereby elevating Human epidermal growth factor receptor 2 (HER2) expression and resistance [51]. Targeting NUPR1 reverses resistance phenotypes, providing a strategy for precision therapy in endocrine-resistant breast cancer.

### Glioblastoma

In glioblastoma (GBM), NUPR1 enhances temozolomide resistance by inhibiting LCN2-mediated iron efflux [83, 85] and by upregulating autophagy-related molecules SNAP25 and multidrug-resistance transporters like MDR1 and MRP1, thereby promoting drug efflux and stress adaptation to establish broad-spectrum resistance [33]. Single-cell transcriptomics and immunohistochemistry reveal high NUPR1 in brain metastases [123], closely correlating with enhanced chemoresistance. Recent evidence indicates that TFP suppresses NUPR1, thereby downregulating SNAP25 and disrupting VAMP8-mediated autophagosome–lysosome fusion, which leads to p62 accumulation, concomitant reductions in lysosomal and DNA-repair proteins including RAD51 and BRCA1/2), and impaired resolution of therapy-induced DNA damage [118]. In parallel, TFP enhances ER Ca2⁺ release and oxidative stress to trigger apoptosis; suppresses LCN2 to promote Fe2⁺ accumulation and ferroptosis; and prevents NUPR1-mediated degradation of p27/p21, thereby restraining cell-cycle progression and proliferation [83].

### Pancreatic cancer

In pancreatic cancer, under gemcitabine stress, NUPR1 is upregulated via the integrated stress response, suppresses caspase-3 activation, and cooperates with autophagy and antioxidant programs to enhance resistance [5, 72]. Inhibiting NUPR1 or the Integrated stress response (ISR) restores apoptosis and gemcitabine sensitivity.

Gemcitabine (Gem) activates the integrated stress response (ISR), driving eIF2α-ATF4-dependent translational reprogramming that upregulates anti-apoptotic and metabolic/antioxidant-stress effectors and thereby establishes an adaptive survival program underpinning chemoresistance in pancreatic ductal adenocarcinoma [72]. Central to ISR is the reversible phosphorylation of eIF2α. NUPR1 directly modulates the eIF2α axis and governs the checkpoint that transitions cells from global translational attenuation to restoration of protein synthesis; loss of NUPR1 sustains phosphorylated eIF2α and impedes translational recovery under stress [5]. In line with this mechanism, pharmacological inhibition of NUPR1 with ZZW-115 exhibits robust cytotoxicity in Gem-resistant PDAC cell lines and attenuates chemoresistance [49, 79], highlighting the NUPR1–eIF2α axis as a tractable therapeutic target for reversing Gem resistance.

### Other cancer

In prostate cancer, NUPR1 mediates docetaxel resistance via activation of the PTEN/AKT/mTOR axis and regulates ubiquitination-related genes to sustain the resistant phenotype [81, 94]. In colorectal cancer, NUPR1-dependent autophagy counteracts oxaliplatin-induced DNA damage, whereas Fat mass and obesity-associated protein (FTO)-mediated m⁶A demethylation of *LCN2* and *FTH1* suppresses ferroptosis and enhances chemoresistance [106]. In clear-cell renal cell carcinoma (ccRCC), FTO-mediated demethylation upregulates NUPR1, activating AKT/mTOR signaling and stemness programs such as *OCT4*, *SOX2*, *NANOG* and *CD44*, thereby promoting self-renewal and sorafenib resistance [39, 66]. In gastric cancer, NUPR1 is activated by Yes-associated protein (YAP), enhances AKT/p21 signaling, promotes Epithelial–mesenchymal transition (EMT), inhibits apoptosis, and increases resistance to 5-fluorouracil (5-FU) and oxaliplatin [44].

Collectively, these findings position NUPR1 as a multidimensional hub of therapy resistance, integrating stress-response programs with cell-death control, autophagy, and metabolic plasticity—thereby offering actionable nodes for combination strategies across cancer types.

## NUPR1-targeted therapeutic strategies

Although NUPR1 exerts pronounced oncogenic and stress-adaptive functions across cancers, the development of small-molecule inhibitors remains limited. As an IDP lacking a stable fold, NUPR1 poses major challenges for structure-based discovery, rendering conventional screening largely ineffective. To overcome this, investigators adopted a ligand-based rational-design strategy, leading to discovery and optimization of the representative NUPR1 inhibitor ZZW-115 [79]. Trifluoperazine (TFP) was first identified as a NUPR1-binding inhibitor; however, central nervous system adverse effects precluded clinical repurposing as an anticancer agent [79]. Building on this, molecular-dynamics simulations and docking delineated key TFP–NUPR1 binding regions. Chemical modification of the TFP scaffold—particularly the piperazine-ring methyl groups—yielded optimized derivatives [79]. Biophysical assays (fluorescence, NMR, circular dichroism) confirmed that ZZW-115 binds NUPR1 with high affinity (K_d ≈ 2.1 μM), inducing local conformational changes in key residues without altering its intrinsically disordered character [79]. Functionally, ZZW-115 showed potent antitumor activity across pancreatic and other solid-tumor cell lines, whereas activity was largely abolished in NUPR1-knockout cells, confirming target specificity [79]. In vivo, ZZW-115 markedly inhibited pancreatic-tumor growth in xenografted mice without neurotoxicity, supporting therapeutic potential [79]. Mechanistically, ZZW-115 acts via dual programs: (i) metabolic dysregulation—driving excessive ROS, increasing oxidized glutathione (GSSG), and downregulating *GPX4*, thereby collapsing antioxidant defenses; and (ii) mitochondrial damage—disrupting mitochondrial integrity in HCC and PDAC cells, promoting abnormal iron accumulation, and triggering ferroptosis [50]. At the level of nuclear transport, ZZW-115 binds the NUPR1 nuclear-localization signal (NLS), competitively blocking interaction with importin Karyopherin subunit alpha-4 (*KPNA4)*, thereby preventing NUPR1 nuclear translocation and impairing DNA-damage repair. Consequently, ZZW-115 potentiates genotoxic agents—including 5-fluorouracil, oxaliplatin, gemcitabine, and γ-irradiation [49].

Despite promising preclinical activity, ZZW-115 significantly interacts with hERG potassium channels [12, 64], potentially causing QT prolongation and cardiotoxicity. To mitigate this liability, the Iovanna group used a high-throughput thermal-shift assay (TSA) to screen the HitFinder library, identifying a new scaffold, AJO, with minimal hERG affinity [64]. Among these, AJO14 emerged as the lead, showing strong affinity for NUPR1, negligible hERG inhibition, and dose-dependent suppression of tumor growth in PDAC mouse models [64]. Subsequent structure–activity relationship (SAR) optimization yielded LZX-2–73, an AJO14-derived analog refined by scaffold modification [64]. Unlike TFP-based inhibitors, this next-generation compound engages multiple NUPR1 sites, functionally inactivates the protein, and induces multiple death programs—including apoptosis, programmed necrosis, and Poly (ADP-ribose) polymerase (PARP)-hyperactivation–mediated parthanatos [64]. These effects coincide with mitochondrial dysfunction and ATP depletion and are reversed by the PARP inhibitor olaparib [64], indicating a mechanism involving *PARP1* overactivation. LZX-2–73 retains potent NUPR1 inhibition (IC₅₀ ≈ 0.4 μM) and demonstrates a favorable cardiac-safety profile in preclinical toxicology [64]. LZX-2–73 combined with sorafenib exhibits pronounced synergy across multiple tumor models. Mechanistically, the combination amplifies reactive oxygen species and lipid peroxidation, lowers the GSH/GSSG ratio, and suppresses the NRF2–xCT–GPX4 antioxidant axis, thereby triggering LDH release and caspase-3/7 activation and culminating in predominantly apoptosis-mediated cell death. These effects sensitize tumors to sorafenib and help overcome resistance, providing an actionable combination strategy—particularly for HCC and PDAC [63].

**Fig. 4:**
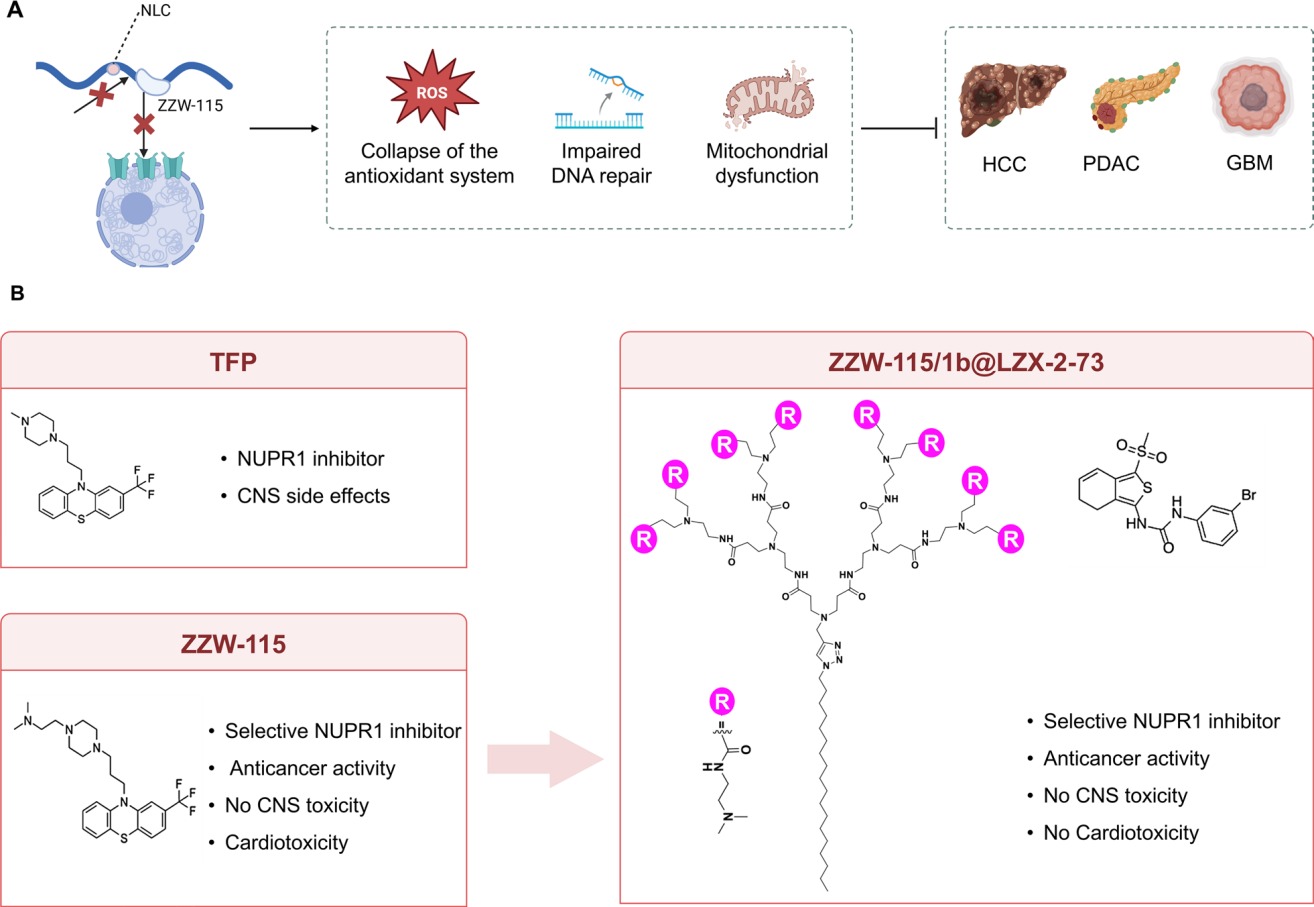
NUPR1-targeted inhibitors, delivery strategies, and antitumor mechanisms. **A** Mechanism of action (exemplified by ZZW-115): ZZW-115 specifically binds to the nuclear localization signal (NLS) of NUPR1, competitively blocking its interaction with the nuclear transport protein KPNA4, thereby inhibiting nuclear translocation and downstream transcriptional activity. Concurrently, it induces antioxidant system collapse, impairs DNA repair, and causes mitochondrial dysfunction, which together enhance tumor cell sensitivity to chemo- and radiotherapy (e.g., 5-FU, oxaliplatin, gemcitabine, γ-radiation). ZZW-115 exhibits inhibitory activity across multiple solid tumors, including HCC, PDAC, and GBM. **B** Inhibitors and Optimization Progress: TFP: The first identified NUPR1 inhibitor, but its clinical translation was limited by central nervous system (CNS) side effects. ZZW-115: A selective NUPR1 inhibitor with potent antitumor activity and minimal CNS toxicity; however, its interaction with the hERG potassium channel suggests a potential risk of cardiotoxicity. LZX-2–73 and nanoformulated ZZW-115/1b@: LZX-2–73, a next-generation multi-site NUPR1 inhibitor derived from AJO14, induces apoptosis, programmed necrosis, and parthanatos with superior preclinical cardiac safety compared to ZZW-115. The amphiphilic dendrimer-based nanoformulation ZZW-115/1b@ reduces hERG interaction, prolongs circulation, enhances tumor accumulation, and synergistically strengthens antitumor efficacy. (Adapted with permission from [62], Creative Commons Attribution NonCommercial License 4.0 (CC BY-NC). This figure is a schematic representation and not drawn to scale. It was created using BioRender based on integrated findings from multiple studies (see “NUPR1-Targeted Therapeutic Strategies” section and References). For specific data or statistical methods, please refer to the original research. *Abbreviations* NLS, nuclear localization signal; KPNA4, karyopherin subunit alpha 4; hERG, human ether-à-go-go-related gene (K⁺) channel; CNS, central nervous system

More recently, precise delivery of ZZW-115 via a self-assembling, dendrimer-based nanoplatform significantly reduced hERG-associated toxicity while enhancing antitumor efficacy [62]. In preclinical PDAC models, fluorinated amphiphilic dendrimer micelles carrying doxorubicin (FAD-Dox) exploit ~ 10-nm size, acid-responsive release, and macropinocytosis-biased uptake to sustain tumor enrichment and achieve deep penetration, yielding more uniform and potent antitumor activity than free or liposomal doxorubicin while improving pharmacokinetics and lowering systemic toxicity—collectively supporting this nanoplatform’s translational promise [60]. An amphiphilic dendrimer (dendrimer-1b) formed self-assembled, drug-loaded nanoparticles ZZW-115/1b@ Encapsulation of ZZW-115—without altering its chemical structure—prevented direct hERG interaction, thereby reducing hERG affinity [62]. In vivo, ZZW-115/1b@ exhibited prolonged circulation, higher tumor accumulation, and robust tumor-growth inhibition in two PDAC xenograft models [62], without detectable cardiac or systemic toxicity. Immunohistochemistry showed reduced Ki-67 positivity and increased caspase-3-mediated apoptosis, indicating antitumor effects associated with cell-cycle arrest and apoptosis induction (Table 2 and Fig. 4).

**Table 2:** NUPR1-targeted therapeutic agents: mechanisms of action and therapeutic potential

Inhibitor	Origin	Mechanism of action	Experimental model	Combination drug	Combination effect	References
Trifluoperazine (TFP)	Antipsychotic phenothiazine	Induces endoplasmic reticulum (ER) stress and apoptosis	PDAC cell-line models	Bortezomib	Potentiates proteasome-inhibitor-induced apoptosis; enhances cancer-cell death	[41]
ZZW-115	TFP-inspired ligand-guided small molecule	Induces mitochondrial dysfunction and ferroptosis	PDAC and HCC xenografts; solid-tumor cell lines	Paclitaxel	Sensitizes to cytotoxic/genotoxic therapies; increases chemosensitivity	[42, 79]
TFP-2HCl/ZZW-115-2HCl	Salt forms (improved solubility) of TFP/ZZW-115	NUPR1 inhibition; reprograms TAMs; reduces T-cell exhaustion; alleviates immunosuppression	HCC models (THP-1, BMDMs, xenografts)	PD-L1 inhibitor (ICB)	Enhances ICB response; improves TME immune activation	[6]
AJO14 and derivatives	Novel synthetic small-molecule scaffold	Inhibit NUPR1 activity	PDAC xenografts (in vivo), multiple cancer cell-line models	Olaparib (PARP inhibitor), Sorafenib	Attenuates cytotoxicity; Sensitizes to sorafenib; boosts ROS/lipid peroxidation while suppressing NRF2–xCT–GPX4	[63, 64]

The AJO14-series × Olaparib combination is a mechanistic rescue (attenuation) rather than a therapeutic synergy, supporting a model of PARP1 overactivation in AJO14-derivative cytotoxicity

TFP, Trifluoperazine; PDAC, Pancreatic ductal adenocarcinoma; HCC, Hepatocellular carcinoma; NUPR1, Nuclear protein 1; TAMs, Tumor-associated macrophages; BMDMs, Bone-marrow-derived macrophages; PD-L1, Programmed death-ligand 1; ICB, Immune checkpoint blockade; TME, Tumor microenvironment; PARP, Poly(ADP-ribose) polymerase; ROS, Reactive oxygen species; NRF2, Nuclear factor erythroid 2-related factor 2; xCT, Cystine/glutamate antiporter (system Xc⁻, SLC7A11); GPX4, Glutathione peroxidase 4; ER, Endoplasmic reticulum; THP-1, THP-1 human monocytic leukemia cell line

## Perspectives and conclusions

Although NUPR1 is widely recognized as a multidimensional regulatory hub in cancer, several key questions require in-depth investigation:

### Insufficient understanding of isoform-specific NUPR1 functions

NUPR1 exists as two isoforms (a and b), yet research has predominantly focused on NUPR1b, leaving isoform-specific functions, differences, and potential cooperativity largely unexplored [28, 90]. Future studies should employ CRISPR–Cas9 editing to generate isoform-resolved knockout/overexpression cell lines and animal models. Integration of transcriptomic, proteomic, and metabolomic analyses will elucidate how distinct isoforms contribute to tumor progression, immune-microenvironment remodeling, and therapeutic resistance, thereby providing a conceptual basis for precise targeting strategies.

### NUPR1-targeted drug development remains early, with pharmacodynamic and safety bottlenecks yet to be overcome

Although small-molecule inhibitors such as ZZW-115 show potent preclinical antitumor activity, off-target hERG potassium-channel interactions raise cardiotoxicity concerns [76, 92]. As a complementary strategy, nanodelivery systems have demonstrated superior toxicity mitigation [11, 110], thereby further enhancing the clinical translational potential of ZZW-115. Future discovery should leverage high-throughput screening and AI-assisted design to identify novel scaffolds, enhancing NUPR1 specificity and affinity while minimizing off-target toxicity. Moreover, structural optimization is needed to improve tumor penetration, bioavailability, and metabolic stability, thereby enhancing translational potential.

### The immunoregulatory role of NUPR1 within the tumor microenvironment remains incompletely defined

While existing studies indicate that NUPR1 modulates TAM polarization and shapes immunosuppressive milieus, its cross-cancer heterogeneity and detailed molecular circuitry remain unclear [15, 74, 109]. Future work should integrate single-cell sequencing, spatial transcriptomics, and proteomics to delineate how NUPR1 interacts with immune subsets such as Tregs, MDSCs, and NK cells, thereby defining dynamic control of immune heterogeneity and immunotherapy responsiveness. Furthermore, prospective validation of NUPR1-inhibitor combinations with immune-checkpoint blockade like anti-PD-L1 may establish a mechanistic basis for precision immunotherapy.

### Comprehensive evaluation of NUPR1’s translational potential is urgently needed

Although accumulating evidence identifies NUPR1 as a hub of tumor stress adaptation, the directionality and magnitude of its effects across tumor types, metabolic states, and immune ecologies—and the underlying effector modules—remain unclear. Notably, isoform divergence, post-translational modifications such as phosphorylation, ubiquitination, and lactylation, and subcellular trafficking may reshape its transcriptional and metabolic outputs through context-specific interactomes that have not yet been causally defined. To this end, future studies should deploy isoform-resolved genetic frameworks coupled with temporally controlled perturbations and spatially resolved sequencing/imaging to construct a dynamic atlas of the “NUPR1–interactome–pathway output” continuum. Mechanistic endpoints ought to include real-time metabolic flux assays, mitochondrial functional readouts, and quantitative metrics of nuclear import–export and DNA damage–repair, with cross-layer validation in patient-derived organoids, immune co-culture systems, and in vivo models to ensure reproducibility and translatability. On the translational front, composite pharmacodynamic surrogates—target engagement, nuclear-translocation blockade, and downstream biomarker responses—should anchor early signal-seeking studies and enable rational combinations with chemo/radiotherapy, cell-death inducers, or immunotherapies. Finally, enrollment stratification schemes grounded in transcriptional signatures, metabolic/PTM markers, and immune composition are needed to delineate indication boundaries and beneficiary subgroups, thereby converting NUPR1 from an “inhibitable” target into a usable and controllable body of clinical evidence.

Future NUPR1 research should integrate deep mechanistic investigation with translational priorities. By leveraging multidisciplinary technologies and multidimensional data integration, the field can accelerate translation from basic discovery to precision oncology, thereby providing a robust scientific foundation and innovative strategies for individualized cancer therapy and precision medicine.

## Data Availability

Not applicable.

## Abbreviations

NUPR1: Nuclear protein 1
PEST: Proline-, glutamic acid-, serine- and threonine-rich
IDP: Intrinsically disordered protein
SMAD: SMA/mothers against decapentaplegic
M2: Alternatively activated macrophage
RelB: Reticuloendotheliosis viral oncogene homolog B
IER3: Immediate early response 3
TFE3: Transcription factor binding to IGHM enhancer 3
ERBB2: Erb-b2 receptor tyrosine kinase 2
TCGA: The cancer genome atlas
HPA: Human protein atlas
PDAC: Pancreatic ductal adenocarcinoma
HCC: Hepatocellular carcinoma
BC: Breast cancer
CRC: Colorectal cancer
NSCLC: Non-small cell lung cancer
PanIN: Pancreatic intraepithelial neoplasia
NAFL: Nonalcoholic fatty liver
NASH: Nonalcoholic steatohepatitis
UPR: Unfolded protein response
PERK: Protein kinase RNA-like ER kinase
ATF4: Activating transcription factor 4
CHOP: C/EBP homologous protein
IRE1: Inositol-requiring enzyme 1
XBP1s: Spliced X-box binding protein 1
ATF6: Activating transcription factor 6
SCAP: SREBP cleavage-activating protein
FASN: Fatty acid synthase
SCD1: Stearoyl-CoA desaturase 1
GRN: Granulin
MAPK: Mitogen-activated protein kinase
HK2: Hexokinase 2
LDHA: Lactate dehydrogenase A
AURKA: Aurora kinase A
ATG: Autophagy-related gene
OSCC: Oral squamous cell carcinoma
LAMP1: Lysosomal-associated membrane protein 1
LC3B-II: Microtubule-associated protein 1A/1B-light chain 3 beta-II
SQSTM1: Sequestosome-1 (p62)
SNAP25: Synaptosomal-associated protein 25
VAMP8: Vesicle-associated membrane protein 8
SNARE: Soluble NSF attachment protein receptor
BCL-2: B-cell lymphoma 2
AhR: Aryl hydrocarbon receptor
CYP: Cytochrome P450
ROS: Reactive oxygen species
LCN2: Lipocalin-2
MYH11: Myosin heavy chain 11
TNBC: Triple-negative breast cancer
FTH1: Ferritin heavy chain 1
AT2: Alveolar type II
OXPHOS: Oxidative phosphorylation
LLPS: Liquid–liquid phase separation
SG: Stress granule
TME: Tumor microenvironment
ECM: Extracellular matrix
TIME: Tumor immune microenvironment
MDSCs: Myeloid-derived suppressor cells
TAMs: Tumor-associated macrophages
VEGF: Vascular endothelial growth factor
PD-L1: Programmed death-ligand 1
CCL2: C–C motif chemokine ligand 2
CSF1: Colony-stimulating factor 1
M1: Classically activated macrophage
H3K18la: Lactylation at histone H3 lysine 18
NSC59984: P73 agonist
L-DOXR: Long-term doxorubicin-resistant
CDKN1A: Cyclin-dependent kinase inhibitor 1A (p21)
RB: Retinoblastoma protein
ESR1: Estrogen receptor 1
RAB31: Ras-related protein Rab-31
HER2: Human epidermal growth factor receptor 2
MDR1/ABCB1: Multidrug resistance protein 1 (P-glycoprotein)
MRP1/ABCC1: Multidrug resistance-associated protein 1
ISR: Integrated stress response
OCT4: Octamer-binding transcription factor 4
SOX2: SRY-box transcription factor 2
NANOG: Homeobox protein NANOG
CD44: Cluster of differentiation 44
YAP: Yes-associated protein
EMT: Epithelial–mesenchymal transition
BC: Breast cancer
PCa: Prostate cancer
G3BP1: Ras GTPase-activating protein-binding protein 1
FTO: Fat mass and obesity-associated protein
GC: Gastric cancer
TFP: Trifluoperazine
GSSG: Oxidized glutathione
NLS: Nuclear localization signal
KPNA4: Karyopherin subunit alpha-4
TSA: Thermal shift assay
SAR: Structure–activity relationship
LZX-2-73: AJO14-derived NUPR1 inhibitor
PARP: Poly(ADP-ribose) polymerase
PARP1: Poly(ADP-ribose) polymerase 1
CNS: Central nervous system
PI3K: Phosphoinositide 3-kinase
AKT: Protein kinase B
mTOR: Mechanistic target of rapamycin
PPARα: Peroxisome proliferator-activated receptor alpha
SREBP1: Sterol regulatory element-binding protein 1
mSREBP1: Mature SREBP1
PINK1: PTEN-induced kinase 1
Parkin: E3 ubiquitin ligase Parkin
ERK1/2: Extracellular signal-regulated kinase 1/2
JNK: C-Jun N-terminal kinase
ARG1: Arginase 1
TREM2: Triggering receptor expressed on myeloid cells 2
VEGFA: Vascular endothelial growth factor A
WTAP: Wilms tumor 1-associating protein
DLBCL: Diffuse large B-cell lymphoma
HUVECs: Human umbilical vein endothelial cells
TEV: Tumor-derived extracellular vesicle
CH25H: Cholesterol 25-hydroxylase
IFNAR1: Interferon alpha/beta receptor 1
IFN1: Type I interferon
NINJ1: Nerve injury-induced protein 1
hERG: Human ether-a-go-go-related gene potassium channel
eIF2α: Eukaryotic initiation factor 2 alpha
3-MA: 3-Methyladenine
RAPA: Rapamycin
5-FU: 5-Fluorouracil
